# Outcome of breath tests in adult patients with suspected small intestinal bacterial overgrowth 

**Published:** 2017

**Authors:** Johanna Mattsson, Maria Teresa Minaya, Milka Monegro, Benjamin Lebwohl, Suzanne K. Lewis, Peter HR Green, Reidun Stenberg

**Affiliations:** 1 *Örebro University Hospital, * *Örebro University, Örebro, Sweden*; 2 *Department of Medicine, Celiac Disease Center, Columbia University College of Physicians and Surgeons, Columbia University Medical Center, New York, USA*; 3 *University Health Care Research Center, School of Health and Medical Sciences, Örebro University, Örebro, Sweden*

**Keywords:** Small intestinal bacterial overgrowth, Lactulose breath test, Glucose breath test, Gastrointestinal symptoms, Adults.

## Abstract

**Aim:**

The aim was to investigate breath test outcomes in patients with suspected SIBO and indicative symptoms of SIBO, diagnosed by breath testing.

**Background:**

Breath testing is used to detect small intestinal bacterial overgrowth (SIBO) by measuring hydrogen and methane produced by intestinal bacteria.

**Methods:**

This retrospective cross sectional study included 311 patients with gastrointestinal symptoms who underwent the breath test for evaluation of SIBO at Celiac Disease Center at Columbia University, New York, in 2014-2015. The patients were divided into two groups based on the physician’s choice: lactulose breath test group (72%) and glucose breath test group (28%). Among them, 38% had a history of celiac disease or non-celiac gluten sensitivity.

**Results:**

In total, 46% had a positive breath test: 18% were positive for methane, 24 % positive for hydrogen and 4% positive for both gases (p=0.014). Also, 50% had a positive lactulose breath result and 37% had a positive glucose breath result (p=0.036). The most common symptom for performing the breath test was bloating and the only clinical symptom that significantly showed a positive glucose breath test was increased gas (p=0.028).

**Conclusion:**

Lactulose breath test was more often positive than glucose breath test. Positivity for hydrogen was more common than methane. Bloating was the most frequently perceived symptom of the patients undergoing the breath test but the only statistically significant clinical symptom for a positive glucose breath test was increased gas. Furthermore, the results showed that there was no significant association between positive breath test result and gender, age, non-celiac gluten sensitivity or celiac disease.

## Introduction

 In small intestinal bacterial overgrowth (SIBO), there is an increased number of bacteria in the small intestine, defined as the microbiological presence of 10^5^ or more colony forming units of bacteria per milliliter of proximal jejunal aspiration (1-5). Patients with SIBO may suffer from abdominal pain, bloating, nausea, constipation, diarrhea and gas (1, 6-9), but may also be asymptomatic (6, 9). The etiology of SIBO is not completely understood since many factors affect the bacterial flora in the intestine (3-5, 8, 10).

The gold standard for diagnosing SIBO is culture of the intestinal fluid (1, 5, 6, 8, 9). Breath tests are used as a surrogate marker for SIBO, which measure hydrogen (H_2_) and methane (CH_4_) in expired breath. These method are inexpensive and non-invasive (8, 10, 11).

The breath test involves the patient drinking a sugar solution of lactulose or glucose. The bacterial fermentation of carbohydrates in the intestine produces H_2 _(5, 10-13) and about 15%-30% of the population are colonized with *Methanobrevibacter smithii*, which converts H_2_ to CH_4 _(14). H_2_ and CH_4_ diffuse into the blood and the lungs where the gases are exhaled (5, 11, 12).

Patients breathe into a breath analyzer every 20 minutes for three hours (5, 10, 11). If either H_2_ or CH_4_ appears in the expired air, it indicates that bacterial fermentation of carbohydrates has taken place, which may be caused by SIBO (10, 12).

Glucose is normally absorbed in the proximal small intestine, and if the patient has bacterial overgrowth, there will be bacterial fermentation before glucose is absorbed, showing rise of H_2_ or CH_4_ in the glucose breath test. A positive lactulose breath test will present two peaks; an early H_2_ or CH_4_ peak, representing the bacterial fermentation of lactulose in the small intestine, and the late peak due to exhaled H_2_ or CH_4_ as a consequence of colonic bacterial metabolism, which is normal (10-12).

Breath tests require a bacterial flora that can metabolize carbohydrate to H_2_, otherwise the test can be falsely negative. The tests can also be falsely positive if the oral bacterial flora is producing H_2 _(13).

The aim of this study was to investigate the breath test outcome in adult patients with suspected SIBO and indicative symptoms of SIBO, diagnosed by breath testing. 

## Methods

**Study design**

This retrospective cross-sectional study was carried out at Celiac Disease Center at Columbia University, New York. Ethical approval was provided by the Ethical committee of Columbia University, New York, IRB-AAAL 4504, Breath testing database.

**Study subjects**

The inclusion criteria for the study were adult patients (over 18 years) who were referred to the Celiac Disease Center at Columbia University to have a breath test referred by their gastroenterologist due to persistent symptoms of diarrhea, constipation, gas, bloating and abdominal pain. The study included 311 patients who underwent the breath test in 2014-2015. 

Exclusion criteria were antibiotic use in the preceding month before the exam, concomitant use of probiotics at least 10 days prior to the exam, patients who chew gum or smoked cigarettes one hour before or during the test, patients who exercised or slept one hour before or during the test, concomitant use of laxatives one week prior to the test, patients who brushed their teeth prior to the test and patients who did not adhere to the breath test preparation diet the day prior to the exam (diet consisting of eggs, chicken, fish, white rice and water).

The study participants ranged from 18 to 91 years in age and the mean age was 47 years, 74% (n=230) were women and 26% (n=81) were men. Totally, 38% (n=117) had a history of celiac disease or non-celiac gluten sensitivity (defined by physicians). The study participants were divided into two groups depending on whether they underwent the lactulose or the glucose breath test.

**Data collection**

Analytical records of 311 patients who underwent the breath test were reviewed. In the analytical records, the patients had reported their symptoms and we also obtained information about the given substrate (lactulose or glucose), gender, age and the outcome of the breath test. The weight changes were defined as unexplained during the last six months and the rest of the symptoms were defined in a period of one month. 

**Breath test**

Before the breath test, the patients were instructed about the restrictions according to the exclusion criteria. Before the test, the patients rinsed their mouth with antiseptic mouthwash. 

The breath test started by collecting a baseline sample where the H_2_ and CH_4_ levels in patient’s breath were examined. Then, the patients drank a solution of lactulose or glucose, based on the physician’s choice. The patients breathed into a breath analyzer (QuinTron, QuinTron Instrument Company, USA) every 20 minutes for three hours and the breath samples were examined. 

A positive lactulose breath test was defined as an increase of either H_2_ or CH_4_ at least 20 ppm (parts per million) within the first 90 minutes, followed by a larger peak. The glucose breath test was positive if H_2_ or CH_4_ increased at least 12 ppm over the lowest preceding value within the test period (15).

**Statistical analysis**

All data were analyzed using IBM SPSS Statistics 23. Comparisons of breath test results, positivity for H_2_ and CH_4_, glucose vs. lactulose breath test and symptoms were performed using a Pearson Chi-square test or a Fisher’s exact test. T-test was used to compare ages. Two-sided P-values of less than 0.05 were considered statistically significant. 

## Results

**Baseline characteristics**

The study participants (n=311) were divided into two groups, 72% (n=224) underwent the lactulose breath test and 28% (n=87) underwent the glucose breath test. Baseline characteristics are listed in Table 1.

**Table 1:** Baseline characteristics of 311 patients who underwent the glucose breath test or the lactulose breath test at Celiac Disease Center, Columbia University

	Glucose breath test (n=87)	Lactulose breath test (n=224)
Age, mean ± SD	47±17	47±17
Gender, n (%)		
Female Age 18-45 Age 46-80 Age 81 and over	74/87(85)37370	156/224(70)68853
Male Age 18-45 Age 46-80 Age 81 and over	13/87(15)571	68/224(30) 40262
Celiac disease/ non-celiac gluten sensitivity Female Male	38/87(44)335	79/224(35) 5920

**Symptoms**

**Figure 1:**
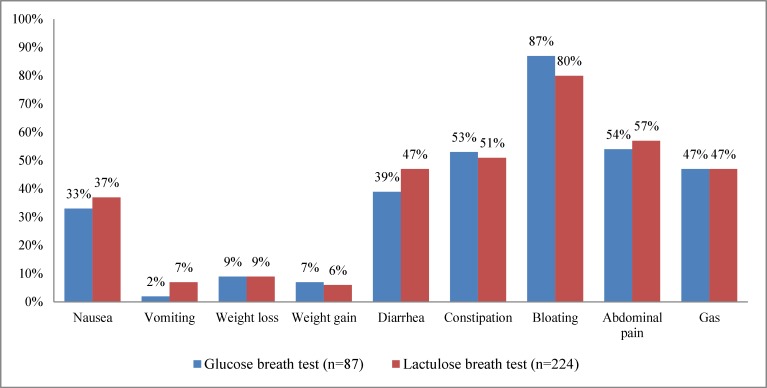
Reported symptoms for patients undergoing the glucose or the lactulose breath test.

The most commonly perceived symptom in both lactulose and glucose breath test group was bloating (80-87%). Other symptoms were abdominal pain (54-57%), constipation (51-53%), increased gas (47%) and diarrhea (39-47%) (Figure 1).

Of the patients who had a positive glucose breath test, 63 % complained of increased gas while among those who had a negative glucose breath test, 38% complained of increased gas (p=0.028). Of the patients who had a positive lactulose breath test, 5% experienced weight loss and of the patients who had a negative lactulose breath test, 13% experienced weight loss (p=0.039). There was no significant difference concerning the other symptoms correlated to the breath test result. 

**Breath test result**

**Figure 2:**
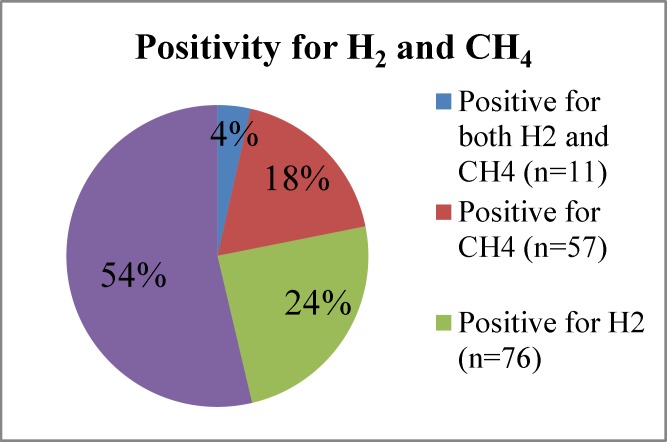
Distribution of positivity for H_2_ and CH_4_, for patients undergoing the glucose or the lactulose breath test (n=311

In total, 37% (32/87) had a positive glucose breath test and 50% (112/224) had a positive lactulose breath test (p=0.036). Altogether, 46% (144/311) had a positive test, including 4% (11/311) positive for both CH_4_ and H_2_, 18% (57/311) positive for CH_4_ and 24% (76/311) positive for H_2_ (p=0.014) (Figure 2).

Of the women who underwent breath testing, 46% (105/230) had a positive test and among the men, 48% (39/81) had a positive breath test (p=0.698). The mean age for a positive breath test was 49 years and the mean age for a negative breath test was 45 years (p=0.104). 

Of the patients who underwent the lactulose breath test and had a history of celiac disease/non-celiac gluten sensitivity, 49% had a positive test while among patients without celiac disease, 50% had a positive test (p=0.928). Of the patients who underwent the glucose breath test and had a history of celiac disease/non-celiac gluten sensitivity, 29% had a positive test while among patients without celiac disease, 43% had a positive test (p=0.182).

## Discussion

In this retrospective cohort study of 311 patients undergoing breath testing, 46% had a positive breath test result. 

At the Celiac Disease Center at Columbia University, the lactulose breath test was more commonly used, as per the preference of the physicians at the center. This study indicated that the lactulose breath test results were more often positive (50%) compared to the glucose breath test (37%). This finding is consistent with other studies that also found that glucose breath test may underdiagnose SIBO since the method fails to diagnose SIBO in the distal part of the small intestine (11, 12, 16). However, the lactulose breath test may overdiagnose SIBO if the patients have a rapid orocecal transit time (11, 14, 17). One improvement in this study would be if all the patients did both the lactulose and the glucose breath test, which would have allowed for direct comparison of these tests. 

It was more common for patients to be positive for H_2_ rather than CH_4_. This is expected since the bacterial fermentation of glucose or lactulose in the small intestine produces H_2_ (5, 10-13); however, not everyone produces CH_4_. Approximately 15-30% of the population are colonized with Methanobrevibacter smithii, which makes them capable of converting H2 to CH_4_ (14).

The most commonly reported symptom in both lactulose and glucose breath test groups was bloating which was also the most common symptom with a positive breath test, although not significantly. 

Patients with a positive glucose breath test were more likely to complain of increased gas than the patients with a negative test. Patients with a negative lactulose breath test result more often experienced weight loss than those with a positive test. Another study demonstrated that bloating, flatus and increased satiety are related to a positive glucose breath test result among patients with inflammatory bowel disease (18).

According to other studies, it seems more likely for women to experience SIBO compared to men (19, 20), but we could not confirm this finding. In addition, there was no statistically significant association between the breath test result and whether the patient had a history of celiac disease/non-celiac gluten sensitivity, indicating that SIBO occurs in both conditions. 

Previous studies have demonstrated that SIBO is associated with increasing age, probably because the elderly have reduced intestinal motility (19, 20). This study does not support this finding since the mean ages for positive and negative breath test were not significantly different. 

The strengths of this study include its sample size and the fact that we had access to the analytical records of all 311 patients. A general limitation of this kind of retrospective studies is that the patients are not randomized, which may affect the results, e.g. if the patients in the two groups (lactulose and glucose) have underlying differences in comorbidity. Future considerations could include randomizing the patients to lactulose versus glucose breath testing in a prospective setting.

In total, 46% of the referred patients had a positive breath test. The most commonly perceived symptom among all participants was bloating. The only symptom significantly associated with a positive glucose breath test result was increased gas (p=0.028). 

This study indicated that the lactulose breath test results are more often positive compared to the glucose breath test. H_2_ positivity was more common compared to CH_4_. Furthermore, the results showed that there was no significant association between positive breath test result and gender, age, non-celiac gluten sensitivity or celiac disease.
